# Advances and Challenges in Protein-Ligand Docking

**DOI:** 10.3390/ijms11083016

**Published:** 2010-08-18

**Authors:** Sheng-You Huang, Xiaoqin Zou

**Affiliations:** 1 Dalton Cardiovascular Research Center, University of Missouri, Columbia, MO 65211, USA; Email: huangshe@missouri.edu; 2 Department of Physics and Astronomy, University of Missouri, Columbia, MO 65211, USA; 3 Department of Biochemistry, University of Missouri, Columbia, MO 65211, USA; 4 Informatics Institute, University of Missouri, Columbia, MO 65211, USA

**Keywords:** protein flexibility, ligand sampling, scoring functions, molecular docking, protein-ligand interactions

## Abstract

Molecular docking is a widely-used computational tool for the study of molecular recognition, which aims to predict the binding mode and binding affinity of a complex formed by two or more constituent molecules with known structures. An important type of molecular docking is protein-ligand docking because of its therapeutic applications in modern structure-based drug design. Here, we review the recent advances of protein flexibility, ligand sampling, and scoring functions—the three important aspects in protein-ligand docking. Challenges and possible future directions are discussed in the Conclusion.

## 1. Introduction

Molecular recognitions including enzyme-substrate, drug-protein, drug-nucleic acid, protein-nucleic acid, and protein-protein interactions play important roles in many biological processes such as signal transduction, cell regulation, and other macromolecular assemblies. Therefore, determination of the binding mode and affinity between the constituent molecules in molecular recognition is crucial to understanding the interaction mechanisms and to designing therapeutic interventions. Due to the difficulties and economic cost of the experimental methods for determining the structures of complexes, computational methods such as molecular docking are desired for predicting putative binding modes and affinities. In molecular docking, based on the protein structures, thousands of possible poses of association are tried and evaluated; the pose with the lowest energy score is predicted as the “best match”, *i.e.*, the binding mode. Since Kuntz and colleagues’ pioneering work [1], significant progress has been made in docking research to improve the computational speed and accuracy. Among them, protein-ligand docking is a particularly vibrant research area because of its importance to structure-based drug design [2–8] and will be the subject of the present review.

**Figure 1:**
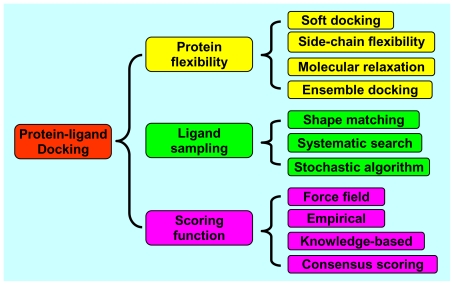
Classification of the methods for protein-ligand docking.

A protein-ligand docking program consists of two essential components, sampling and scoring. Sampling refers to the generation of putative ligand binding orientations/conformations near a binding site of a protein and can be further divided into two aspects, ligand sampling and protein flexibility. Scoring is the prediction of the binding tightness for individual ligand orientations/conformations with a physical or empirical energy function. The top orientation/conformation, namely the one with the lowest energy score, is predicted as the binding mode. Here, we will review the recent advances in protein-ligand docking on three important aspects: protein flexibility, ligand sampling, and scoring function, as illustrated in Figure 1. Challenges and future directions will also be discussed.

## 2. Protein Flexibility

Ligand binding commonly induces protein conformational changes (referred to as “induced fit”), which range from local rearrangements of side-chains to large domain motions. Due to the large size and many degrees of freedom of proteins, their flexibility may be the most challenging issue in molecular docking. Current methods to account for protein flexibility can be grouped into four categories: soft docking, side-chain flexibility, molecular relaxation, and protein ensemble docking [9–14].

### 2.1. Soft Docking

Soft docking is the simplest method which considers protein flexibility implicitly. It works by allowing for a small degree of overlap between the ligand and the protein through softening the interatomic van der Waals interactions in docking calculations [15,16]. The advantages of soft docking are its computational efficiency and easiness for implementation. However soft docking can account for only small conformational changes.

### 2.2. Side-Chain Flexibility

Many of the early attempts to incorporate certain protein conformational changes into molecular docking focused on side-chain flexibility, in which backbones are kept fixed and side-chain conformations are sampled. One of the earliest studies is the ligand docking algorithm developed by Leach, in which discrete side-chain flexibility is included by using a rotamer library [17]. Since then, researchers have proposed many improved techniques to incorporate continuous or discrete side-chain flexibility in ligand docking [18–24].

### 2.3. Molecular Relaxation

The third type of methods account for protein flexibility by firstly using rigid-body docking to place the ligand into the binding site and then relaxing the protein backbone and side-chain atoms nearby. Specifically, the initial rigid-body docking allows for atomic clashes between the protein and the placed ligand orientations/conformations in order to consider the protein conformational changes. Then, the formed complexes are relaxed or minimized by Monte Carlo (MC), Molecular Dynamic simulations, or other methods [25,26]. The advantage of the molecular relaxation method is the inclusion of certain backbone flexibility in addition to the side-chain conformational changes. However, compared to the side-chain flexibility methods, the relaxation method is more demanding for the scoring function because it involves not only the side-chain movement but also the more challenging backbone sampling, thereby inaccuracies in the scoring function may lead to artifacts (e.g., improper backbone torsions) in the relaxed protein conformations. Moreover, the relaxation method is time-consuming.

### 2.4. Docking of Multiple Protein Structures

The most widely-used type of methods for incorporating protein flexibility utilize an ensemble of protein structures to represent different possible conformational changes [9–14]. One of the earliest studies was done by Knegtel *et al.* [27], in which an averaged energy grid was constructed by combining the energy grids generated from individual experimentally-determined protein structures using a weighting scheme, followed by standard ligand docking. Osterberg *et al.* extended the method to AutoDock [28] with a larger ensemble consisting of 21 different conformations of the HIV-1 protease [29]. The averaging nature of the method may miss the geometric accuracy of the protein. Claussen *et al.* developed a docking program FlexE to dock ligands into an ensemble of protein structures [30], in which the similar segments of the protein structures in the ensemble are aligned and merged while the dissimilar segments are used to combinatorially create new possible protein conformations for docking. In Wei *et al.*’s algorithm [31], a protein was decomposed into a rigid part and several flexible parts according to the crystal structures of the protein in the ensemble. For a given ligand placement, only the best-fit local conformer was kept for each flexible part in the protein, assuming the flexible regions move independently. The selected local conformers were joined with the rigid part to form the best-fit protein conformation. Compared to FlexE, this algorithm is significantly faster and scales linearly rather than exponentially with the protein flexibility. However, the algorithm may rely on the quality of ligand orientational/conformational sampling, namely whether proper initial ligand placement is included. Huang and Zou developed a fast ensemble docking algorithm by treating the protein conformational ensemble as an additional dimension to the traditional six degrees of freedom (three translational plus three rotational) for ligand energy optimization [32,33]. The algorithm is almost as fast as single docking whereas keeping the accuracy of sequential docking. The ensemble docking algorithm is not used for generating new protein structures, but instead for selecting the induced-fit structure from a given protein ensemble. Following a similar procedure, Abagyan and colleagues expanded Huang and Zou’s algorithm to create ICM’s ensemble docking algorithm, referred to as four-dimensional (4D) docking [34]. In addition to experimental structures such as NMR structures or crystal structures bound of the protein with different ligands, ensembles of protein conformations generated by molecular dynamic simulations, Monte Carlo simulations, or structure prediction have also been used to account for protein flexibility [35–43].

## 3. Ligand Sampling

Ligand sampling is the most basic element in protein-ligand docking. Given a protein target, the sampling algorithm generates putative ligand orientations/conformations (*i.e.*, poses) around the chosen binding site of the protein. The binding site can be the experimentally determined active site, a dimer interface or other site of interest. Ligand sampling is the most successful area being developed in protein-ligand docking. Roughly, there are three types of ligand sampling algorithms: shape matching, systematic search, and stochastic algorithms.

### 3.1. Shape Matching

The shape matching method is one of the simplest sampling algorithms which is often used in the early stages of the docking process or in the first step of other more advanced ligand sampling methods. It places the ligand using the criterion that the molecular surface of the placed ligand must complement the molecular surface of the binding site on the protein. The six degrees of freedom (three translational and three rotational) of the ligand allow for many possible ligand binding orientations. Therefore, how to quickly place the ligand in the binding site with a good shape complementarity is the goal of the shape matching algorithm. Examples of docking programs that use shape matching include DOCK [1], FRED [44], FLOG [45], EUDOC [46], LigandFit [47], Surflex [48], MS-DOCK [49], and MDock [32,33]. The major advantage of shape matching is its computational efficiency. However, the conformation of the ligand is normally fixed during the shape matching process. Therefore, flexible-ligand docking with the shape matching method is usually performed by docking an ensemble of pre-generated ligand conformations into the protein [50], followed by merging/reranking of the docked poses from different docking runs according to their energy scores (see below).

### 3.2. Systematic Search

Systematic search algorithms are normally used for flexible-ligand docking, which generate all possible ligand binding conformations by exploring all degrees of freedom of the ligand. There are three types of systematic search methods: exhaustive search, fragmentation and conformational ensemble.

The most straightforward systematic algorithms are exhaustive search methods, in which flexible-ligand docking is performed by systematically rotating all possible rotatable bonds of the ligand at a given interval. Despite its sampling completeness for ligand conformations, the number of the combinations can be huge with the increase of the rotatable bonds. Therefore, to make the docking process practical, geometric/chemical constrains are normally applied to the initial screening of ligand poses, and the filtered ligand conformations are further subject to the more accurate refinement/optimization procedures. Glide [51,52] and FRED [44] are two typical examples of this type of hierarchical sampling methods.

In fragmentation methods, the ligand is first divided into different rigid parts/fragments. Then, the ligand binding conformation is incrementally grown by placing one fragment at a time in the binding site or by docking all the fragments into the binding site and linking them covalently. DOCK [53], LUDI [54], FlexX [55], ADAM [56], and eHiTs [57] are example docking programs that use fragmentation methods.

In conformational ensemble methods [50], ligand flexibility is represented by rigidly docking an ensemble of pre-generated ligand conformations with other programs such as OMEGA (OpenEye Scientific Inc, NM). Then, ligand binding modes from different docking runs are collected and ranked according to their binding energy scores. Examples of the conformational ensemble methods for docking include FLOG [45], DOCK3.5 [58], PhDOCK [59], MS-DOCK [49], MDock [32,33], and Q-Dock [60].

### 3.3. Stochastic Algorithms

In stochastic algorithms, ligand binding orientations and conformations are sampled by making random changes to the ligand at each step in both the conformational space and the translational/rotational space of the ligand, respectively. The random change will be accepted or rejected according to a probabilistic criterion. There are four types of stochastic algorithms: Monte Carlo (MC) methods, evolutionary algorithms (EA), Tabu search methods, and swarm optimization (SO) methods.

In a Monte Carlo method, the probability to accept a random change is calculated by using the following Boltzmann probability function:

(1) $$P∼\text{exp}\left[\frac{-(E_{1}-E_{0})}{k_{\text{B}}T}\right]$$

where *E*_0_ and *E*_1_ stand for the energy scores of the ligand before and after the random change, respectively, *k*_B_ is the Boltzmann constant, and *T* is the absolute temperature of the system. The docking programs that use the MC methods include DockVision [61], ICM [18], QXP [62], Prodock [63], and MCDOCK [64].

Evolutionary algorithms (EAs) search for the correct ligand binding mode based on the idea from the evolutionary process in biological systems. The most popular type of EAs is the genetic algorithms (GAs). GOLD [65,66], AutoDock [28], DIVALI [67], DARWIN [68], MolDock [69], PSI-DOCK [70], FLIPDock [42], Lead finder [71], and EADock [72] are the examples that have implemented evolution algorithms.

In Tabu search methods, the probability of acceptance depends on the previously explored areas in the conformational space of the ligand. The random change will be rejected if the RMSD between the current ligand binding conformation and any of the previously recorded solutions is less than a cutoff; otherwise, the random change will be accepted. Example docking programs are PRO_LEADS [73] and PSI-DOCK [70].

Swarm optimization (SO) algorithms attempt to find an optimal solution in a search space by modeling swarm intelligence. In the method, movements of a ligand mode through the search space are guided by the information of the best positions of its neighbors. Examples of docking programs that use swarm optimization algorithms include SODOCK [74], Tribe-PSO [75], PSO@Autodock [76], and PLANTS [77].

## 4. Scoring Functions

The scoring function is a key element of a protein-ligand docking algorithm, because it directly determines the accuracy of the algorithm [78–82]. Speed and accuracy are the two important aspects of a scoring function. An ideal scoring function would be both computationally efficient and reliable. Numerous scoring functions have been developed in the past decades and can be grouped into three basic categories according to their methods of derivation: force field, empirical, and knowledge-based scoring functions.

### 4.1. Force Field Scoring Functions

Force field (FF) scoring functions [28,83,84] are based on decomposition of the ligand binding energy into individual interaction terms such as van der Waals (VDW) energies, electrostatic energies, bond stretching/bending/torsional energies, etc., using a set of derived force-field parameters such as AMBER [85] or CHARMM [86,87] force fields. One of the major challenges in FF scoring functions is how to account for the solvent effect. The simplest method is to use a distance-dependent dielectric constant *ɛ* (*r_ij_*) such as the force field scoring function in DOCK [84]:

(2) $$E=\sum_{i}\sum_{j}\left(\frac{A_{ij}}{r_{ij}^{12}}-\frac{B_{ij}}{r_{ij}^{6}}+\frac{q_{i}q_{j}}{ɛ(r_{ij})r_{ij}}\right)$$

where *r_ij_* stands for the distance between protein atom *i* and ligand atom *j*, *A_ij_* and *B_ij_* are the VDW parameters, and *q_i_* and *q_j_* are the atomic charges. *ɛ* (*r_ij_*) is usually set to 4*r_ij_*, reflecting the screening effect of water on electrostatic interactions.

The most rigorous FF methods are to treat water molecules explicitly such as FEP and TI (see [88] for review). However, these methods, together with their simplified approaches such as LIEPROFEC, and OWFEG are computationally expensive [88]. To reduce the computational expense, accelerated methods have been developed while preserving the reasonable accuracy by treating water as a continuum dielectric medium. The Poisson-Boltzmann/surface area (PB/SA) models [89–99] and the generalized-Born/surface area (GB/SA) models [100–111] are typical examples of such implicit solvent models.

In addition to the challenge on solvent effect, how to accurately account for entropic effect is an even more severe challenge for FF scoring functions. Moreover, whether the free energy of ligand binding can be decomposed into a linear combination of individual interaction terms without calculating the partition function (“ensemble average”) also remains in question, referred to as the nonadditive problem.

### 4.2. Empirical Scoring Functions

In empirical scoring functions, the binding energy score of a complex is calculated by summing up a set of weighted empirical energy terms such as VDW energy, electrostatic energy, hydrogen bonding energy, desolvation term, entropy term, hydrophobicity term, *etc*.

(3) $$\Delta G=\sum_{i}W_{i}\cdot \Delta G_{i}$$

where {Δ*G_i_*} represent individual empirical energy terms, and the corresponding coefficients {*W_i_*} are determined by reproducing the binding affinity data of a training set of protein-ligand complexes with known three-dimensional structures, using least squares fitting [112–121]. Compared to the force field scoring functions, the empirical scoring functions are normally much more computationally efficient due to their simple energy terms. However, the general applicability of an empirical scoring function depends on the training set due to the nature of its fitting to known binding affinities of its training set. With the rapid increase in the number of crystal structures of diverse protein-ligand complexes with known binding affinities, a general empirical scoring function could be developed by training on the binding constants of thousands of protein-ligand complexes. GlideScore [51,52], PLP [119,120], SYBYL/F-Score [55], LigScore [118], LUDI [115,117], SCORE [116], X-Score [121], ChemScore [114], MedusaScore [122], AIScore [123], and SFCscore [124] are examples of empirical scoring functions.

### 4.3. Knowledge-Based Scoring Functions

The potential parameters of knowledge-based scoring functions are directly derived from the structural information in experimentally determined protein-ligand complexes [125–128]. The principle behind knowledge-based scoring functions is the potential of mean force [129], which is defined by the inverse Boltzmann relation [130–133]

(4) $$w(r)=-k_{\text{B}}T\text{ln}[\rho (r)/\rho^{*}(r)]$$

where *k*_B_ is the Boltzmann constant, *T* is the absolute temperature of the system, *ρ*(*r*) is the number density of the protein-ligand atom pair at distance *r* in the training set, and ρ^*^(*r*) is the pair density in a reference state where the interatomic interactions are zero. After the potential parameters *w*(*r*) are derived, the energy of ligand binding for a given complex is simply the sum of the interaction terms for all the protein-ligand atom pairs in the complex. Based on the early idea of Tanaka and Scheraga [125], a number of knowledge-based scoring functions have been developed for protein-ligand interactions. Compared to the force field and empirical scoring functions, the knowledge-based scoring functions offer a good balance between accuracy and speed. Namely, because the potentials in Equation. (4) are extracted from a large number of structures rather than attempting to reproduce the known affinities by fitting, the knowledge-based scoring functions are relatively robust and general [134–137]. Their pairwise characteristic also enables the scoring process to be as fast as empirical scoring functions.

As the ideal reference state is inaccessible for complicated systems like proteins [132], one major challenge for knowledge-based scoring functions is the calculation for the afore-mentioned reference state; based on the methods knowledge-based scoring functions can be classified into three categories: traditional atom-randomized reference state, corrected reference state, and circumventing the reference state.

Traditional methods to approximate the reference state are randomization of the atoms in the training set. Examples include DrugScore [138,139], SMoG [140,141], BLEEP [142,143], GOLD/ASP [144], MScore [145], and KScore[146]. The disadvantage of the atom-randomization approximation is the neglection of the effects of excluded volume, interatomic connectivity, *etc.* [132]. Methods like PMF [134,135] and DFIRE [147] have introduced correction terms for the reference state. Yet, binding mode prediction and virtual database screening are main problems for most knowledge-based scoring functions as a result of the reference state problem. To circumvent the long-standing reference state problem, Huang and Zou have developed a physics-based iterative method and derived the ITScore scoring function, which has been extensively tested with multiple benchmarks for binding mode prediction, affinity prediction and virtual screening [136,137,148].

Other challenges for knowledge-based scoring functions include extension of the pairwise interactions to many-body interactions to account for hydrogen bonding and other directional interactions, development of an accurate method for entropic calculations [148], *etc*.

### 4.4. Consensus Scoring

Consensus scoring is not really a specific type of scoring function but a technique in protein-ligand docking [149]. It improves the probability of finding a correct solution by combining the scoring information from multiple scoring functions in hopes of balancing out the errors of the individual scoring functions. Therefore, the main issue in consensus scoring is how to make the combination rule for individual scores so that the true binders can be discriminated from others according to the consensus rule [150,151]. MultiScore and X-Cscore are two examples of consensus scoring methods [121,152].

### 4.5. Clustering and Entropy-Based Scoring Methods

In addition to consensus scoring, another technique to improve the performances of scoring functions is clustering-based scoring methods, which incorporate the entropic effects by dividing generated ligand binding modes into different clusters [169–171]. The entropic contribution in each cluster is measured by the configurational space covered by the ligand poses or the number of the ligand poses in the cluster. One restriction in clustering-based scoring methods is that its performance depends on the ligand sampling protocol that is used, *i.e.*, it is docking program-dependent. These methods in combination with ligand conformational sampling using AutoDock have significantly improved binding mode prediction [148,169–171].

## 5. Conclusion and Discussions

We have reviewed three important aspects of protein-ligand docking: protein flexibility, ligand sampling, and scoring functions. Rapid advances in the last two decades have almost solved the ligand sampling issue. Although equal or even more efforts have been paid to scoring function development, entropy and desolvation effects remain the two major challenging issues for current scoring functions, particularly for the force field scoring functions. Speed and accuracy are the two important characteristics of a scoring function. Because of the rapid increase in computing power, how to improve the accuracy is the future direction for scoring function development. In contrast to ligand sampling and scoring functions which have been extensively studied for more than two decades, protein flexibility has only been addressed recently because of the difficulty resulting from the enormous degrees of freedom and the limitation of the computing power. The development of computational methods for protein flexibility is still in its infancy and thereby remains one of the major future directions in protein-ligand docking. Finally, how to evaluate different docking programs and scoring functions is another active area [153–157]. Although many comparison studies for docking and scoring have been published [158–166], publicly available docking benchmarks such as DUD (http://dud.docking.org/) [167,168] and CSAR (http://www.csardock.org/) are extremely valuable for systematic and consistent evaluation and improvement of new and existing docking algorithms.

## References

[1] Kuntz ID Blaney JM Oatley SJ Langridge R Ferrin TE A geometric approach to macromolecule-ligand interactions J. Mol. Biol 1982 161 269 288 7154081 10.1016/0022-2836(82)90153-x

[2] Brooijmans N Kuntz ID Molecular recognition and docking algorithms Annu. Rev. Biophys. Biomol. Struct 2003 32 335 373 12574069 10.1146/annurev.biophys.32.110601.142532

[3] Halperin I Ma B Wolfson H Nussinov R Principles of docking: An overviewof search algorithms and a guide to scoring functions Proteins 2002 47 409 443 12001221 10.1002/prot.10115

[4] Shoichet BK McGovern SL Wei B Irwin JJ Lead discovery using molecular docking Curr. Opin. Chem. Biol 2002 6 439 446 12133718 10.1016/s1367-5931(02)00339-3

[5] Kitchen DB Decornez H Furr JR Bajorath J Docking and scoring in virtual screening for drug discovery: Methods and applications Nat. Rev. Drug Discov 2004 3 935 948 15520816 10.1038/nrd1549

[6] Muegge I Rarey M Small molecule docking and scoring Rev. Comput. Chem 2001 17 1 60

[7] Sousa SF Fernandes PA Ramos MJ Protein-ligand docking: Current status and future challenges Proteins 2006 65 15 26 16862531 10.1002/prot.21082

[8] Kolb P Ferreira RS Irwin JJ Shoichet BK Docking and chemoinformatic screens for new ligands and targets Curr. Opin. Biotech 2009 20 429 436 19733475 10.1016/j.copbio.2009.08.003 PMC2766606

[9] Carlson HA Protein flexibility is an important component of structure-based drug discovery Curr. Pharm. Des 2002 8 1571 1578 12052201 10.2174/1381612023394232

[10] Carlson HA McCammon JA Accommodating protein flexibility in computational drug design Mol. Pharmacol 2000 57 213 218 10648630

[11] Teodoro ML Kavraki LE Conformational flexibility models for the receptor in structure based drug design Curr. Pharm. Des 2003 9 1635 1648 12871062 10.2174/1381612033454595

[12] Teague SJ Implications of protein flexibility for drug discovery Nat. Rev. Drug Discov 2003 2 527 541 12838268 10.1038/nrd1129

[13] Cozzini P Kellogg GE Spyrakis F Abraham DJ Costantino G Emerson A Fanelli F Gohlke H Kuhn LA Morris GM Target flexibility: An emerging consideration in drug discovery and design J. Med. Chem 2008 51 6237 6255 18785728 10.1021/jm800562d PMC2701403

[14] Totrov M Abagyan R Flexible ligand docking to multiple receptor conformations: A practical alternative Curr. Opin. Struct. Biol 2008 18 178 184 18302984 10.1016/j.sbi.2008.01.004 PMC2396190

[15] Jiang F Kim SH Soft docking: Matching of molecular surface cubes J. Mol. Biol 1991 219 79 102 2023263 10.1016/0022-2836(91)90859-5

[16] Ferrari AM Wei BQ Costantino L Shoichet BK Soft docking and multiple receptor conformations in virtual screening J. Med. Chem 2004 47 5076 5084 15456251 10.1021/jm049756p PMC1413506

[17] Leach AR Ligand docking to proteins with discrete side-chain flexibility J. Mol. Biol 1994 235 345 356 8289255 10.1016/s0022-2836(05)80038-5

[18] Abagyan R Totrov M Kuznetzov D ICM – a new method for protein modeling and design: Applications to docking and structure prediction from the distorted native conformation J. Comput. Chem 1994 15 488 506

[19] Desmet J Wilson IA Joniau M De Maeyer M Lasters I Computation of the binding of fully flexible peptides to proteins with flexible side-chains FASEB J 1997 11 164 172 9039959 10.1096/fasebj.11.2.9039959

[20] Schaffer L Verkhivker GM Predicting structural effects in HIV-1 protease mutant complexes with flexible ligand docking and protein side-chain optimization Proteins 1998 33 295 310 9779795 10.1002/(sici)1097-0134(19981101)33:2<295::aid-prot12>3.0.co;2-f

[21] Schnecke V Kuhn LA Virtual screening with solvation and ligand-induced complementarity Perspect. Drug Discov. Des 2000 20 171 190

[22] Frimurer TM Peters GH Iversen LF Andersen HS Moller NP Olsen OH Ligand-induced conformational changes: Improved predictions of ligand binding conformations and affinities Biophys. J 2003 84 2273 2281 12668436 10.1016/S0006-3495(03)75033-4 PMC1302794

[23] Meiler J Baker D ROSETTALIGAND: Protein-small molecule docking with full side-chain flexibility Proteins 2006 65 538 584 16972285 10.1002/prot.21086

[24] Nabuurs SB Wagener M de Vlieg J A flexible approach to induced fit docking J. Med. Chem 2007 50 6507 6518 18031000 10.1021/jm070593p

[25] Apostolakis J Pluckthun A Caflisch A Docking small ligands in flexible binding sites J. Comput. Chem 1998 19 21 37

[26] Davis IW Baker D ROSETTALIGAND docking with full ligand and receptor flxibility J. Mol. Biol 2009 385 381 392 19041878 10.1016/j.jmb.2008.11.010

[27] Knegtel RM Kuntz ID Oshiro CM Molecular docking to ensembles of protein structures J. Mol. Biol 1997 266 424 440 9047373 10.1006/jmbi.1996.0776

[28] Morris GM Goodsell DS Halliday RS Huey R Hart WE Belew RK Olson AJ Automated docking using a Lamarckian genetic algorithm and an empirical binding free energy function J. Comput. Chem 1998 19 1639 1662

[29] Osterberg F Morris GM Sanner MF Olson AJ Goodsell DS Automated docking to multiple target structures: Incorporation of protein mobility and structural water heterogeneity in AutoDock Proteins 2002 46 34 40 11746701 10.1002/prot.10028

[30] Claussen H Buning C Rarey M Lengauer T FlexE: Efficient molecular docking considering protein structure variations J. Mol. Biol 2001 308 377 395 11327774 10.1006/jmbi.2001.4551

[31] Wei BQ Weaver LH Ferrari AM Matthews BW Shoichet BK Testing a flexible-receptor docking algorithm in a model binding site J. Mol. Biol 2004 337 1161 1182 15046985 10.1016/j.jmb.2004.02.015

[32] Huang S-Y Zou X Ensemble docking of multiple protein structures: Considering protein structural variations in molecular docking Proteins 2007 66 399 421 17096427 10.1002/prot.21214

[33] Huang S-Y Zou X Efficient molecular docking of NMR structures: Application to HIV-1 protease Protein Sci 2007 16 43 51 17123961 10.1110/ps.062501507 PMC2222846

[34] Bottegoni G Kufareva I Totrov M Abagyan R Four-dimensional docking: A fast and accurate account of discrete receptor flexibility in ligand docking J. Med. Chem 2009 52 397 406 19090659 10.1021/jm8009958 PMC2662720

[35] Broughton HB A method for including protein flexibility in protein-ligand docking: Improving tools for database mining and virtual screening J. Mol. Graph. Model 2000 18 247 257 11021541 10.1016/s1093-3263(00)00036-x

[36] Carlson HA Masukawa KM Rubins K Bushman FD Jorgensen WL Lins RD Briggs JM McCammon JA Developing a dynamic pharmacophore model for HIV-1 integrase J. Med. Chem 2000 43 2100 2114 10841789 10.1021/jm990322h

[37] Meagher KL Carlson HA Incorporating protein flexibility in structure-based drug discovery: Using HIV-1 protease as test case J. Am. Chem. Soc 2004 126 13276 13281 15479081 10.1021/ja0469378

[38] Lin JH Perryman AL Schames JR McCammon JA Computational drug design accommodating receptor flexibility: The relaxed complex scheme J. Am. Chem. Soc 2002 124 5632 5633 12010024 10.1021/ja0260162

[39] Zavodszky MI Lei M Thorpe MF Day AR Kuhn LA Modeling correlated main-chain motions in proteins for flexible molecular recognition Proteins 2004 57 243 261 15340912 10.1002/prot.20179

[40] Cavasotto CN Abagyan RA Protein flexibility in ligand docking and virtual screening to protein kinases J. Mol. Biol 2004 337 209 225 15001363 10.1016/j.jmb.2004.01.003

[41] Sherman W Day T Jacobson MP Friesner RA Farid R Novel procedure for modeling ligand/receptor induced fit effects J. Med. Chem 2006 49 534 553 16420040 10.1021/jm050540c

[42] Zhao Y Sanner MF FLIPDock: Docking flexible ligands into flexible receptors Proteins 2007 68 726 737 17523154 10.1002/prot.21423

[43] May A Zacharias M Protein-ligand docking accounting for receptor side chain and global flexibility in normal modes: Evaluation on kinase inhibitor cross docking J. Med. Chem 2008 51 3499 3506 18517186 10.1021/jm800071v

[44] McGann MR Almond HR Nicholls A Grant JA Brown FK Gaussian docking functions Biopolymers 2003 68 76 90 12579581 10.1002/bip.10207

[45] Miller MD Kearsley SK Underwood DJ Sheridan RP FLOG: A system to select quasi-flexible ligands complementary to a receptor of known three-dimensional structure J. Comput. Aided Mol. Des 1994 8 153 174 8064332 10.1007/BF00119865

[46] Pang YP Perola E Xu K Prendergast FG EUDOC: A computer program for identification of drug interaction sites in macromolecules and drug leads from chemical databases J. Comput. Chem 2001 22 1750 1771 12116409 10.1002/jcc.1129

[47] Venkatachalam CM Jiang X Oldfield T Waldan M LigandFit: A novel method for the shape-directed rapid docking of ligands to protein active sites J. Mol. Graph. Model 2003 21 289 307 12479928 10.1016/s1093-3263(02)00164-x

[48] Jain AN Surflex: Fully automatic molecular docking using a molecular similarity-based search engine J. Med. Chem 2003 46 499 511 12570372 10.1021/jm020406h

[49] Sauton N Lagorce D Villoutreix B Miteva M MS-DOCK: Accurate multiple conformation generator and rigid docking protocol for multi-step virtual ligand screening BMC Bioinformatics 2008 9 184 18402678 10.1186/1471-2105-9-184 PMC2373571

[50] Lorber DM Shoichet BK Hierarchical docking of databases of multiple ligand conformations Curr. Top. Med. Chem 2005 5 739 749 16101414 10.2174/1568026054637683 PMC1364474

[51] Friesner RA Banks JL Murphy RB Halgren TA Klicic JJ Mainz DT Repasky MP Knoll EH Shelley M Perry JK Shaw DE Francis P Shenkin PS Glide: A new approach for rapid, accurate docking and scoring. 1. Method and assessment of docking accuracy J. Med. Chem 2004 47 1739 1749 15027865 10.1021/jm0306430

[52] Halgren TA Murphy RB Friesner RA Beard HS Frye LL Pollard WT Banks JL Glide: A new approach for rapid, accurate docking and scoring. 2. Enrichment factors in database screening J. Med. Chem 2004 47 1750 1759 15027866 10.1021/jm030644s

[53] Ewing TJA Kuntz ID Critical evaluation of search algorithms for automated molecular docking and database screening J. Comput. Chem 1997 18 1175 1189

[54] Bohm HJ The computer program LUDI: A new method for the de novo design of enzyme inhibitors J. Comput. Aided Mol. Des 1992 6 61 78 1583540 10.1007/BF00124387

[55] Rarey M Kramer B Lengauer T Klebe G A fast flexible docking method using an incremental construction algorithm J. Mol. Biol 1996 261 470 489 8780787 10.1006/jmbi.1996.0477

[56] Mizutani MY Tomioka N Itai A Rational automatic search method for stable docking models of protein and ligand J. Mol. Biol 1994 243 310 326 7932757 10.1006/jmbi.1994.1656

[57] Zsoldos Z Reid D Simon A Sadjad BS Johnson AP eHiTS: An innovative approach to the docking and scoring function problems Curr. Protein Pept. Sci 2006 7 421 435 17073694 10.2174/138920306778559412

[58] Lorber DM Shoichet BK Flexible ligand docking using conformational ensembles Protein Sci 1998 7 938 950 9568900 10.1002/pro.5560070411 PMC2143983

[59] Joseph-McCarthy D Thomas BEIV Belmarsh M Moustakas D Alvarez JC Pharmacophore-based molecular docking to account for ligand flexibility Proteins 2003 51 172 188 12660987 10.1002/prot.10266

[60] Brylinski M Skolnick J Q-Dock: Low-resolution flexible ligand docking with pocket-specific threading restraints J. Comput. Chem 2008 29 1574 1588 18293308 10.1002/jcc.20917 PMC2726574

[61] Hart TN Read RJ A multiple-start Monte Carlo docking method Proteins 1992 13 206 222 1603810 10.1002/prot.340130304

[62] McMartin C Bohacek RS QXP: Powerful, rapid computer algorithms for structure-based drug design J. Comput. Aided Mol. Des 1997 11 333 344 9334900 10.1023/a:1007907728892

[63] Trosset JY Scheraga HA Prodock: Software package for protein modeling and docking J. Comput. Chem 1999 20 412 427

[64] Liu M Wang S MCDOCK: A Monte Carlo simulation approach to the molecular docking problem J. Comput. Aided Mol. Des 1999 13 435 451 10483527 10.1023/a:1008005918983

[65] Jones G Willett P Glen RC Molecular recognition of receptor sites using a genetic algorithm with a description of desolvation J. Mol. Biol 1995 245 43 53 7823319 10.1016/s0022-2836(95)80037-9

[66] Jones G Willett P Glen RC Leach AR Taylor R Development and validation of a genetic algorithm for flexible docking J. Mol. Biol 1997 267 727 748 9126849 10.1006/jmbi.1996.0897

[67] Clark KP Flexible ligand docking without parameter adjust-ment across four ligand-receptor complexes J. Comput. Chem 1995 16 1210 1226

[68] Taylor JS Burnett RM DARWIN: A program for docking flexible molecules Proteins 2000 41 173 191 10966571

[69] Thomsen R Christensen MH MolDock: A new technique for highaccuracy molecular docking J. Med. Chem 2006 49 3315 3321 16722650 10.1021/jm051197e

[70] Pei J Wang Q Liu Z Li Q Yang KL Lai L PSI-DOCK: Towards highly efficient and accurate flexible ligand docking Proteins 2006 62 934 946 16395666 10.1002/prot.20790

[71] Stroganov OV Novikov FN Stroylov VS Kulkov V Chilov GG Lead finder: An approach to improve accuracy of protein-ligand docking, binding energy estimation, and virtual screening J. Chem. Inf. Model 2008 48 2371 2385 19007114 10.1021/ci800166p

[72] Grosdidier A Zoete V Michielin O EADock: Docking of small molecules into protein active sites with a multiobjective evolutionary optimization Proteins 2007 67 1010 1025 17380512 10.1002/prot.21367

[73] Baxter CA Murray CW Clark DE Westhead DR Eldridge MD Flexible docking using Tabu search and an empirical estimate of binding affinity Proteins 1998 33 367 382 9829696

[74] Chen H-M Liu B-F Huang H-L Hwang S-F Ho S-Y SODOCK: Swarm optimization for highly flexible protein-ligand docking J. Comput. Chem 2007 28 612 623 17186483 10.1002/jcc.20542

[75] Chen K Li T Cao T Tribe-PSO: A novel global optimization algorithm and its application in molecular docking Chemom. Intell. Lab. Syst 2006 82 248 259

[76] Namasivayam V Gunther R PSO@Autodock: A fast flexible molecular docking program based on swarm intelligence Chem. Biol. Drug. Des 2007 70 475 484 17986206 10.1111/j.1747-0285.2007.00588.x

[77] Korb O Stutzle T Exner TE PLANTS: Application of ant colony optimization to structure-based drug design Ant Colony Optimization and Swarm Intelligence, 5th International Workshop Brussels, Belgium 4–7 September, 2006 247 258

[78] Gohlke H Klebe G Statistical potentials and scoring functions applied to protein-ligand binding Curr. Opin. Struct. Biol 2001 11 231 235 11297933 10.1016/s0959-440x(00)00195-0

[79] Schulz-Gasch T Stahl M Scoring functions for protein-ligand interactions: A critical perspective Drug Discov. Today: Tech 2004 1 231 239 10.1016/j.ddtec.2004.08.004 24981490

[80] Jain AN Scoring functions for protein-ligand docking Curr. Protein Pept. Sci 2006 7 407 420 17073693 10.2174/138920306778559395

[81] Rajamani R Good AC Ranking poses in structure-based lead discovery and optimization: Current trends in scoring function development Curr. Opin. Drug. Discov. Devel 2007 10 308 315 17554857

[82] Gilson MK Zhou HX Calculation of protein-ligand binding affinities Annu. Rev. Biophys. Biomol. Struct 2007 36 21 42 17201676 10.1146/annurev.biophys.36.040306.132550

[83] Huang N Kalyanaraman C Irwin JJ Jacobson MP Molecular mechanics methods for predicting protein-ligand binding J. Chem. Inf. Model 2006 46 243 253 16426060 10.1021/ci0502855

[84] Meng EC Shoichet BK Kuntz ID Automated docking with grid-based energy approach to macromolecule-ligand interactions J. Comput. Chem 1992 13 505 524

[85] Weiner PK Kollman PA AMBER – assisted model building with energy refinementla general program for modeling molecules and their interactions J. Comput. Chem 1981 2 287 303

[86] Nilsson L Karplus M Empirical energy functions for energy minimization and dynamics of nucleic acids J. Comput. Chem 1986 7 591 616

[87] Brooks BR Bruccoleri RE Olafson BD States DJ Swaminathan S Karplus M CHARMM – a programm for macromolecular energy, minimization, and dynamics calculations J. Comput. Chem 1983 4 187 217

[88] Wang W Donini O Reyes CM Kollman PA Biomolecular simulations: Recent developments in force fields, simulations of enzyme catalysis, protein-ligand, protein-protein, and protein-nucleic acid noncovalent interactions Annu. Rev. Biophys. Biomol. Struct 2001 30 211 243 11340059 10.1146/annurev.biophys.30.1.211

[89] Rocchia W Sridharan S Nicholls A Alexov E Chiabrera A Honig B Rapid grid-based construction of the molecular surface and the use of induced surface charge to calculate reaction field energies: Applications to the molecular systems and geometric objects J. Comput. Chem 2002 23 128 137 11913378 10.1002/jcc.1161

[90] Grant JA Pickup BT Nicholls A A smooth permittivity function for Poisson-Boltzmann solvation methods J. Comput. Chem 2001 22 608 640

[91] Baker NA Sept D Joseph S Holst MJ McCammon JA Electrostatics of nanosystems: Application to microtubules and the ribosome Proc. Natl. Acad. Sci. USA 2001 98 10037 10041 11517324 10.1073/pnas.181342398 PMC56910

[92] Wei BQ Baase WA Weaver LH Matthews BW Shoichet BK A model binding site for testing scoring functions in molecular docking J. Mol. Biol 2002 322 339 355 12217695 10.1016/s0022-2836(02)00777-5

[93] Wang J Morin P Wang W Kollman PA Use of MM-PBSA in reproducing the binding free energies to HIV-1 RT of TIBO derivatives and predicting the binding mode to HIV-1 RT of efavirenz by docking and MM-PBSA J. Am. Chem. Soc 2001 123 5221 5230 11457384 10.1021/ja003834q

[94] Kuhn B Gerber P Schulz-Gasch T Stahl M Validation and use of the MM-PBSA approach for drug discovery J. Med. Chem 2005 48 4040 4048 15943477 10.1021/jm049081q

[95] Kuhn B Kollman PA Binding of a diverse set of ligands to avidin and strepavidin: An accurate quantitative prediction of their relative affinities by a combination of molecular mechanics and continuum solvent models J. Med. Chem 2000 43 3786 3791 11020294 10.1021/jm000241h

[96] Pearlman DA Evaluating the molecular mechanics poisson-boltzmann surface area free energy method using a congeneric series of ligands to p38 MAP kinase J. Med. Chem 2005 48 7796 7807 16302819 10.1021/jm050306m

[97] Sims PA Wong CF McCammon JA A computational model of binding thermodynamics: The deisgn of cyclin-dependent kinase 2 inhibitors J. Med. Chem 2003 46 3314 3325 12852762 10.1021/jm0205043

[98] Huang D Caflisch A Efficient evaluation of binding free energy using continuum electrostatics solvation J. Med. Chem 2004 47 5791 5797 15509178 10.1021/jm049726m

[99] Thompson DC Humblet C Joseph-McCarthy D Investigation of MM-PBSA rescoring of docking poses J. Chem. Inf. Model 2008 48 1081 1091 18465849 10.1021/ci700470c

[100] Still WC Tempczyk A Hawley RC Hendrickson T Semianalytical treatment of solvation for molecular mechanics and dynamics J. Am. Chem. Soc 1990 112 6127 6129

[101] Zou X Sun Y Kuntz ID Inclusion of solvation in ligand binding free energy calculations using the generalized-Born model J. Am. Chem. Soc 1999 121 8033 8043

[102] Liu H-Y Kuntz ID Zou X Pairwise GB/SA scoring function for structure-based drug design J. Phys. Chem. B 2004 108 5453 5462

[103] Liu H-Y Zou X Electrostatics of ligand binding: Parametrization of the generalized born model and comparison with the Poisson-Boltzmann approach J. Phys. Chem. B 2006 110 9304 9313 16671749 10.1021/jp060334w PMC2716126

[104] Liu H-Y Grinter SZ Zou X Multiscale generalized born modeling of ligand binding energies for virtual database screening J. Phys. Chem. B 2009 113 11793 11799 19678651 10.1021/jp901212t PMC2763608

[105] Majeux N Scarsi M Apostolakis J Ehrhardt C Caflisch A Exhaustive docking of molecular fragments with electrostatic solvation Proteins 1999 37 88 105 10451553

[106] Cecchini M Kolb P Majeux N Caflisch A Automated docking of highly flexible ligands by genetic algorithms: A critical assessment J. Comput. Chem 2004 25 412 422 14696075 10.1002/jcc.10384

[107] Huang D Luthi U Kolb P Edler K Cecchini M Audetat S Barberis A Caflisch A Discovery of cell-permeable non-peptide inhibitors of beta-secretase by high-throughput docking and continuum electrostatics calculations J. Med. Chem 2005 48 5108 5111 16078830 10.1021/jm050499d

[108] Cho AE Wendel JA Vaidehi N Kekenes-Huskey PM Floriano WB Maiti PK Goddard WA II The MPSim-Dock hierarchical docking algorithm: Application to the eight trypsin inhibitor cocrystals J. Comput. Chem 2005 26 48 71 15529328 10.1002/jcc.20118

[109] Ghosh A Rapp CS Friesner RA Generalized Born model based on a surface integral formulation J. Phys. Chem. B 1998 102 10983 10990

[110] Lyne PD Lamb ML Saeh JC Accurate prediction of the relative potencies of members of a series of kinase inhibitors using molecular docking and MM-GBSA scoring J. Med. Chem 2006 49 4805 4808 16884290 10.1021/jm060522a

[111] Guimaraes CRW Cardozo M MM-GB/SA rescoring of docking poses in structure-based lead optimization J. Chem. Inf. Model 2008 48 958 970 18422307 10.1021/ci800004w

[112] Jain AN Scoring noncovalent protein-ligand interactions: A continuous differentiable function tuned to compute binding affinities J. Comput.-Aided Mol. Des 1996 10 427 440 8951652 10.1007/BF00124474

[113] Head RD Smythe ML Oprea TI Waller CL Green SM Marshall GR Validate a new method for the receptor-based prediction of binding affinities of novel ligands J. Am. Chem. Soc 1996 118 3959 3969

[114] Eldridge MD Murray CW Auton TR Paolini GV Mee RP Empirical scoring functions: I. The development of a fast empirical scoring function to estimate the binding affinity of ligands in receptor complexes J. Comput.-Aided Mol. Des 1997 11 425 445 9385547 10.1023/a:1007996124545

[115] Böhm HJ The development of a simple empirical scoring function to estimate the binding constant for a protein-ligand complex of known three-dimensional structure J. Comput.-Aided Mol. Des 1994 8 243 256 7964925 10.1007/BF00126743

[116] Wang R Liu L Lai L Tang Y SCORE: A new empirical method for estimating the binding affinity of a protein-ligand complex J. Mol. Model 1998 4 379 394

[117] Böhm HJ Prediction of binding constants of ptotein ligands: A fast method for the polarization of hits obtained from de novo design or 3D database search programs J. Comput.-Aided Mol. Des 1998 12 309 323 9777490 10.1023/a:1007999920146

[118] Krammer A Kirchhoff PD Jiang X Venkatachalam CM Waldman M LigScore: A novel scoring function for predicting binding affinities J. Mol. Graph. Model 2005 23 395 407 15781182 10.1016/j.jmgm.2004.11.007

[119] Gehlhaar DK Verkhivker GM Rejto PA Sherman CJ Fogel DB Freer ST Molecular recognition of the inhibitor AG-1343 by HIV-1 Protease: Conformationally flexible docking by evolutionary programming Chem. Biol 1995 2 317 324 9383433 10.1016/1074-5521(95)90050-0

[120] Gehlhaar DK Bouzida D Rejto PA Parrill L Reddy MR Rational Drug Design: Novel Methodology and Practical Applications American Chemical Society Washington, DC, USA 1999 719 292 311

[121] Wang R Lai L Wang S Further development and validation of empirical scoring functions for structure-based binding affinity prediction J. Comput.-Aided Mol. Des 2002 16 11 26 12197663 10.1023/a:1016357811882

[122] Yin S Biedermannova L Vondrasek J Dokholyan NV MedusaScore: An accurate force-field based scoring function for virtual drug screening J. Chem. Inf. Model 2008 48 1656 1662 18672869 10.1021/ci8001167 PMC2665000

[123] Raub S Steffen A Kämper A Marian CM AIScore – Chemically diverse empirical scoring function employing quantum chemical binding energies of hydrogen-bonded complexes J. Chem. Inf. Model 2008 48 1492 1510 18597446 10.1021/ci7004669

[124] Sotriffer CA Sanschagrin P Matter H Klebe G SFCscore: Scoring functions for affinity prediction of protein-ligand complexes Proteins 2008 73 395 419 18442132 10.1002/prot.22058

[125] Tanaka S Scheraga HA Medium- and long-range interaction parameters between amino acids for predicting three-dimensional structures of proteins Macromolecules 1976 9 945 950 1004017 10.1021/ma60054a013

[126] Miyazawa S Jernigan RL Estimation of effective interresidue contact energies from protein crystal structures: Quasi-chemical approximation Macromolecules 1985 18 534 552

[127] Sippl MJ Calculation of conformational ensembles from potentials of mean force J. Mol. Biol 1990 213 859 883 2359125 10.1016/s0022-2836(05)80269-4

[128] Verkhivker G Appelt K Freer ST Villafranca JE Empirical free energy calculations of ligand-protein crystallographic complexes. I. Knowledge-based ligand-protein interaction potentials applied to the prediction of human immunodeficiency virus 1 protease binding affinity Protein Eng 1995 8 677 691 8577696 10.1093/protein/8.7.677

[129] Huang S-Y Zou X Mean-force scoring functions for protein-ligand binding Annu. Rep. Comput. Chem 2010 6 281 296

[130] Thomas PD Dill KA An iterative method for extracting energy-like quantities from protein structures Proc. Natl. Acad. Sci. USA 1996 93 11628 11633 8876187 10.1073/pnas.93.21.11628 PMC38109

[131] Koppensteiner WA Sippl MJ Knowledge-based potentials – Back to the roots Biochemistry (Moscow) 1998 63 247 252 9526121

[132] Thomas PD Dill KA Statistical potentials extracted from protein structures: How accurate are they? J. Mol. Biol 1996 257 457 469 8609636 10.1006/jmbi.1996.0175

[133] McQuarrie DA Statistical Mechanics Harper Collins Publishers New York, NY, USA 1976

[134] Muegge I Martin YC A general and fast scoring function for protein-ligand interactions: A simplified potential approach J. Med. Chem 1999 42 791 804 10072678 10.1021/jm980536j

[135] Muegge I PMF scoring revisited J. Med. Chem 2006 49 5895 5902 17004705 10.1021/jm050038s

[136] Huang S-Y Zou X An iterative knowledge-based scoring function to predict protein-ligand interactions: I. Derivation of interaction potentials J. Comput. Chem 2006 27 1866 1875 16983673 10.1002/jcc.20504

[137] Huang S-Y Zou X An iterative knowledge-based scoring function to predict protein-ligand interactions: II. Validation of the scoring function J. Comput. Chem 2006 27 1876 1882 16983671 10.1002/jcc.20505

[138] Gohlke H Hendlich M Klebe G Knowledge-based scoring function to predict protein-ligand interactions J. Mol. Biol 2000 295 337 356 10623530 10.1006/jmbi.1999.3371

[139] Velec HFG Gohlke H Klebe G DrugScore^CSD^-knowledge-based scoring function derived from small molecule crystal data with superior recognition rate of near-native ligand poses and better affinity prediction J. Med. Chem 2005 48 6296 6303 16190756 10.1021/jm050436v

[140] DeWitte RS Shakhnovich EI SMoG: de Novo design method based on simple, fast, and accutate free energy estimate. 1. Methodology and supporting evidence J. Am. Chem. Soc 1996 118 11733 11744

[141] Ishchenko AV Shakhnovich EI Small molecule growth 2001 (SMoG2001): An improved knowledge-based scoring function for protein-ligand interactions J. Med. Chem 2002 45 2770 2780 12061879 10.1021/jm0105833

[142] Mitchell JBO Laskowski RA Alex A Thornton JM BLEEP – Potential of mean force describing protein-ligand interactions: I. Generating potential J. Comput. Chem 1999 20 1165 1176

[143] Mitchell JBO Laskowski RA Alex A Forster MJ Thornton JM BLEEP – Potential of mean force describing protein-ligand interactions: II. Calculation of binding energies and comparison with experimental data J. Comput. Chem 1999 20 1177 1185

[144] Mooij WT Verdonk ML General and targeted statistical potentials for protein-ligand interactions Proteins 2005 61 272 287 16106379 10.1002/prot.20588

[145] Yang CY Wang RX Wang SM M-score: A knowledge-based potential scoring function accounting for protein atom mobility J. Med. Chem 2006 49 5903 5911 17004706 10.1021/jm050043w

[146] Zhao X Liu X Wang Y Chen Z Kang L Zhang H Luo X Zhu W Chen K Li H Wang X Jiang H An improved PMF scoring function for universally predicting the interactions of a ligand with protein, DNA, and RNA J. Chem. Inf. Model 2008 48 1438 1447 18553962 10.1021/ci7004719

[147] Zhang C Liu S Zhu Q Zhou Y A knowledge-based energy function for protein-ligand, protein-protein, and protein-DNA complexes J. Med. Chem 2005 48 2325 2335 15801826 10.1021/jm049314d

[148] Huang S-Y Zou X Inclusion of solvation and entropy in the knowledge-based scoring function for protein-ligand interactions J. Chem. Inf. Model 2010 50 262 273 20088605 10.1021/ci9002987 PMC3199178

[149] Charifson PS Corkery JJ Murcko MA Walters WP Consensus scoring: A method for obtaining improved hit rates from docking databases of three-dimensional structures into proteins J. Med. Chem 1999 42 5100 5109 10602695 10.1021/jm990352k

[150] Wang R Wang S How does consensus scoring work for virtual library screening? An idealized computer experiment J. Chem. Inf. Comput. Sci 2001 41 1422 1426 11604043 10.1021/ci010025x

[151] Clark RD Strizhev A Leonard JM Blake JF Matthew JB Consensus scoring for ligand/protein interactions J. Mol. Graph. Model 2002 20 281 295 11858637 10.1016/s1093-3263(01)00125-5

[152] Terp GE Johansen BE Christensen IT Jorgensen FS A new concept for multidimensional selection of ligand conformations (MultiSelect) and multidimensional scoring (MultiScore) of protein-ligand binding affinities J. Med. Chem 2001 44 2333 2343 11428927 10.1021/jm001090l

[153] Cole JC Murray CW Nissink JWM Taylor RD Taylor R Comparing protein-ligand docking programs is difficult Proteins 2005 60 325 332 15937897 10.1002/prot.20497

[154] Chen H Lyne PD Giordanetto F Lovell T Li J On evaluating molecular-docking methods for pose prediction and enrichment factors J. Chem. Inf. Model 2006 46 401 415 16426074 10.1021/ci0503255

[155] Jain AN Nicholls A Recommendations for evaluation of computational methods J. Comput.-Aided Mol. Des 2008 22 133 139 18338228 10.1007/s10822-008-9196-5 PMC2311385

[156] Hawkins PCD Warren GL Skillman AG Nicholls A How to do an evaluation: Pitfalls and traps J. Comput.-Aided Mol. Des 2008 22 179 190 18217218 10.1007/s10822-007-9166-3 PMC2270916

[157] Kirchmair J Markt P Distinto S Wolber G Langer T Evaluation of the performance of 3D virtual screening protocols: RMSD comparisons, enrichment assessments, and decoy selectionsWhat can we learn from earlier mistakes J. Comput.-Aided Mol. Des 2008 22 213 228 18196462 10.1007/s10822-007-9163-6

[158] Wang R Lu Y Wang S Comparative evaluation of 11 scoring functions for molecular docking J. Med. Chem 2003 46 2287 2303 12773034 10.1021/jm0203783

[159] Stahl M Rarey M Detailed analysis of scoring functions for virtual screening J. Med. Chem 2001 44 1035 1042 11297450 10.1021/jm0003992

[160] Ferrara P Gohlke H Price DJ Klebe G Brooks CL III Assessing scoring functions for protein-ligand interactions J. Med. Chem 2004 47 3032 3047 15163185 10.1021/jm030489h

[161] Bissantz C Folkers G Rognan D Protein-based virtual screening of chemical databases 1. Evaluation of different docking/scoring combinations J. Med. Chem 2000 43 4759 4767 11123984 10.1021/jm001044l

[162] Perola E Walters WP Charifson PS A detailed comparison of current docking and scoring methods on systems of pharmaceutical relevance Proteins 2004 56 235 249 15211508 10.1002/prot.20088

[163] Warren GL Andrews CW Capelli A-M Clarke B LaLonde J Lambert MH Lindvall M Nevins N Semus SF Senger S Tedesco G Wall ID Woolven JM Peishoff CE Head MS A critical assessment of docking programs and scoring functions J. Med. Chem 2006 49 5912 5931 17004707 10.1021/jm050362n

[164] Kellenberger E Rodrigo J Muller P Rognan D Comparative evaluation of eight docking tools for docking and virtual screening accuracy Proteins 2004 57 225 242 15340911 10.1002/prot.20149

[165] Bursulaya BD Totrov M Abagyan R Brooks CL III Comparative study of several algorithms for flexible ligand docking J. Comput. Aided Mol. Des 2003 17 755 763 15072435 10.1023/b:jcam.0000017496.76572.6f

[166] Kim R Skolnick J Assessment of programs for ligand binding affinity prediction J. Comput. Chem 2008 29 1316 1331 18172838 10.1002/jcc.20893 PMC2702145

[167] Huang N Shoichet BK Irwin JJ Benchmarking sets for molecular docking J. Med. Chem 2006 49 6789 6801 17154509 10.1021/jm0608356 PMC3383317

[168] Irwin JJ Shoichet BK Mysinger MM Huang N Colizzi F Wassam P Cao Y Automated docking screens: A feasibility study J. Med. Chem 2009 52 5712 5720 19719084 10.1021/jm9006966 PMC2745826

[169] Ruvinsky AM Calculations of protein-ligand binding entropy of relative and overall molecular motions J. Comput.-Aided Mol. Des 2007 21 361 370 17503189 10.1007/s10822-007-9116-0

[170] Chang MW Belew RK Carroll KS Olson AJ Goodsell DS Empirical entropic contributions in computational docking: Evaluation in APS reductase complexes J. Comput. Chem 2008 29 1753 1761 18351616 10.1002/jcc.20936 PMC3052286

[171] Lee J Seok C A statistical rescoring scheme for protein-ligand docking: Consideration of entropic effect Proteins 2008 70 1074 1083 18076034 10.1002/prot.21844

